# A modular cell-based biosensor using engineered genetic logic circuits to detect and integrate multiple environmental signals

**DOI:** 10.1016/j.bios.2012.08.011

**Published:** 2013-02-15

**Authors:** Baojun Wang, Mauricio Barahona, Martin Buck

**Affiliations:** a Department of Mathematics, 6M50 Huxley Building, Imperial College London, London, SW7 2AZ, UK; b Department of Life Sciences, Imperial College London, UK; c Centre for Synthetic Biology and Innovation, Imperial College London, UK

**Keywords:** Cellular biosensor, Genetic circuits, Logic gates, Cell programming, Synthetic biology

## Abstract

Cells perceive a wide variety of cellular and environmental signals, which are often processed combinatorially to generate particular phenotypic responses. Here, we employ both single and mixed cell type populations, pre-programmed with engineered modular cell signalling and sensing circuits, as processing units to detect and integrate multiple environmental signals. Based on an engineered modular genetic AND logic gate, we report the construction of a set of scalable synthetic microbe-based biosensors comprising exchangeable sensory, signal processing and actuation modules. These cellular biosensors were engineered using distinct signalling sensory modules to precisely identify various chemical signals, and combinations thereof, with a quantitative fluorescent output. The genetic logic gate used can function as a biological filter and an amplifier to enhance the sensing selectivity and sensitivity of cell-based biosensors. In particular, an *Escherichia coli* consortium-based biosensor has been constructed that can detect and integrate three environmental signals (arsenic, mercury and copper ion levels) via either its native two-component signal transduction pathways or synthetic signalling sensors derived from other bacteria in combination with a cell-cell communication module. We demonstrate how a modular cell-based biosensor can be engineered predictably using exchangeable synthetic gene circuit modules to sense and integrate multiple-input signals. This study illustrates some of the key practical design principles required for the future application of these biosensors in broad environmental and healthcare areas.

## Graphical abstract

**Graphical abstract:**

**Figure:**
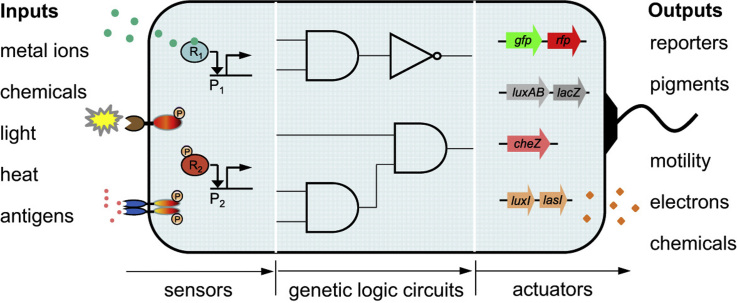

**Highlights:**

► Modular cellular biosensors comprising exchangeable genetic sensors, logic circuits and actuators. ► A set of biosensors for toxic metals and bacterial signalling molecules. ► Genetic logic circuits functioning as biological filters and amplifiers to enhance sensing selectivity and sensitivity. ► A triple-input AND logic gated sensor using multiple cellular consortia.

## 1 Introduction

**Fig. 1:**
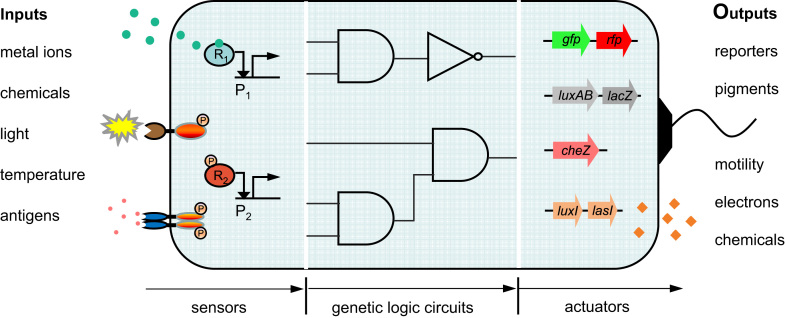
Architecture of a synthetic modular cell-based biosensor. The cellular sensor comprises three interconnected and exchangeable modules, i.e. the input sensors, the internal genetic information processing circuits and the output actuators. The cells are engineered using various natural or synthetic sensors such as sensor kinases or intracellular receptor proteins to detect environmental signals and genetic circuits such as AND and NOT logic gates to modulate and integrate these multiple input signals. The programmed cells can then initiate customized responses by activating different output genes according to the logic decision transmitted upstream. Adapted with permission from Wang and Buck (2012).

Bacterial cells live in an ever changing environment and must therefore be equipped with specific genetically-encoded sensors and signalling networks to continuously perceive and react to the various environmental signals. Analogous to a typical electromechanical sensor, a cellular signalling network normally consists of three interconnected modules – the input sensors, internal processing and regulatory circuits and output actuators to allow signal sensing and timely adaptations in cell physiology (Lim, 2010; Wang and Buck, 2012) (Fig. 1). The input sensors are receptors, either embedded in the cell membrane (e.g. sensor kinases) or located freely in the cytoplasm (e.g. ligand responsive allosteric proteins) with which the cell can detect various extra- or intra-cellular signals, such as chemical molecules, metal ions, light, heat or antigens, and transduce them into differential gene transcriptional levels. Downstream gene regulatory networks process and integrate such signals combinatorially for a logic decision to be made, mimicking the digital logic circuit in electronic circuitry. Decisions are signified by changes in the expression of output actuators: the relevant proteins and chemicals responsible for the final phenotypic changes in motility, growth and morphology etc.

Due to the inherent modular architecture of sensory systems, bacterial cells can be viewed as programmable living biosensors in which the three component modules are exchangeable (Fig. 1). For example, specific synthetic sensors can be developed from either the host's own genetic repertoire or that of other bacterial species with more relevant specialisations in sensing capabilities in order to detect for example particular environmental contaminants or disease-related signals. Informed by the advanced sensing capabilities of many environmental microbes, already a number of single-input bacterial biosensors (van der Meer and Belkin, 2010) have been constructed to detect various toxic pollutant such as arsenic (Joshi et al., 2009; Stocker et al., 2003; Trang et al., 2005), xylene and toluene (Paitan et al., 2004), DNT explosive (De Las Heras et al., 2008) and the human pathogen *Pseudomonas aeruginosa* (Saeidi et al., 2011) with fluorescence, luminescence or colorimetric pigments as outputs. However, these biosensors have been typically developed utilizing only the cell native sensory elements without additional layers of genetic processing and regulation, and thus can only detect one type of signal rather than a number of signals in a predefined combination. Instead, synthetic genetic logic circuits can be introduced through linking various cellular sensors and genetic actuators, to achieve customized cellular responses (Kobayashi et al., 2004) and sensing abilities (Khalil and Collins, 2010) in a designed and logical manner. In particular, multi-input cellular biosensors are essential for recognizing complex conditions, such as the cancer microenvironment normally specified by several signals in combination, where an enhanced sensing specificity and accuracy of the output response are necessary. For example, several genetic logic AND gates have been constructed to link inputs to pathogenicity-related signal responsive promoters (Nissim and Bar-Ziv, 2010), microRNAs (Xie et al., 2011) or proteins (Culler et al., 2010), and the output to a therapeutic suicide gene in order to achieve highly specific *in vivo* sensing and killing of diseased mammalian cells. These proof-of-concept examples demonstrate the great potential for using biological logic circuits to customize cell sensing and signalling for many useful applications (Khalil and Collins, 2010; Wang and Buck, 2012; Weber and Fussenegger, 2012).

Here, we employ both single cell type and multiple cell type populations, pre-programmed with engineered modular cell signalling and sensing circuits, as processing units to sense and integrate multiple environmental contamination associated signals. A modular, tightly-controlled and hypersensitive genetic AND gate was recently constructed in *Escherichia coli* that can integrate two genetic transcriptional inputs simultaneously (Wang et al., 2011). In the present work, we have engineered several *E. coli*-based logic-gated cellular biosensors by connecting the AND gate two inputs to a set of synthetic sensors for detecting and integrating the levels of arsenic, mercury, copper, zinc ions and bacterial quorum sensing molecules in an aqueous environment leading to a quantitative fluorescent output. The designed genetic logic circuits can act as a biological filter and an amplifier to enhance the sensing selectivity and sensitivity of whole cell-based biosensors. Moreover, a triple-input AND logic gated biosensor comprising two cell consortia was also constructed which can sense and integrate three environmental signals (As^3+^, Hg^2+^and Cu^2+^ levels) via a synthetic cell–cell communication module driving the cooperation between the two cell populations.

## 2 Materials and methods

### 2.1 Bacterial strains and growth conditions

Plasmid cloning work and characterisation of the circuit constructs were all performed in *E. coli* TOP10 strain. Cells were cultured in LB (Luria-Bertani Broth) media (10 g/L peptone, 5 g/L NaCl, 5 g/L yeast extract). The antibiotic concentrations used were 50 μg/ml for kanamycin and 50 μg/ml for ampicillin. Cells inoculated from single colonies on freshly streaked plates were grown overnight in 5 ml LB in sterile 20 ml universal tubes at 37 °C with shaking (200 rpm). Overnight cultures were diluted into pre-warmed LB media at OD_600_=0.025 for the day cultures, which were induced and grown for 6 h at 37 °C prior to analysis, unless otherwise indicated. For the fluorescence assay by fluorometry, diluted cultures were loaded into a 96 well microplate (Greiner Bio-One, chimney black, flat clear bottom) by a repetitive pipette and induced with 5 μl (for single input induction) or 10 μl (for double input induction) or 15 μl (for triple input induction) inducers of varying concentrations to a final volume of 200 μl per well by a multichannel pipette. The microplate was covered with a transparent lid to counteract evaporation and incubated in the fluorometer (BMG FLUOstar) with continuous shaking (200 rpm, linear mode) between each cycle of repetitive measurements. All the chemical reagents used were analytical grade and purchased from Sigma Aldrich.

### 2.2 Plasmid circuit construction

Plasmid construction and DNA manipulations were performed following standard molecular biology techniques. The arsenic (J23101-rbs32-*arsR*-B0015-*P*_arsR_) and mercury (J23115-rbs32-*merR*-B0015-*P*_merT_) sensor fragments, *hrpR-*B0015 and *hrpS-*B0015 fragments and *hrpL* promoter were synthesised by GENEART and have been designed following the BioBrick standard (http://biobricks.org), i.e., eliminating the four restriction sites (EcoRI, XbaI, SpeI and PstI) exclusive for this standard by synonymous codon exchange and flanking with prefix and suffix sequences containing the appropriate restriction sites and RBS. The constitutive promoters J23101 and J23115 (http://partsregistry.org) were used to drive gene expression continuously as indicated. The double terminator B0015 was used to terminate gene transcription in all cases. The copper responsive *P*_cusC_, zinc sensitive *P*_zraP_ and cadmium responsive *P*_zntA_ promoters were amplified by colony PCR from the *E. coli* MG1655 genome with EcoRI/SpeI sites. The 3OC_6_HSL sensor (*P*_tet_-rbs34-*luxR*-B0015-*P*_luxI_, BBa_F2620 (Canton et al., 2008)), *gfp* (green fluorescent protein, BBa_E0840) and *rfp* (red fluorescent protein, BBa_I13507) reporters and *luxI* gene (BBa_ K081008) were obtained from the Registry of Standard Biological Parts (http://partsregistry.org). Plasmid pSB3K3 (Shetty et al., 2008) (p15A ori, Kan^r^) was used to carry the six single-input biosensors. The six chemical sensor fragments (cut by EcoRI/SpeI) and the *gfp* or *P. syringae hrpR*/*hrpS* expression constructs (cut by XbaI/PstI) were ligated into pSB3K3 (cut by EcoRI/PstI) following a three way ligation protocol in one pool. Plasmid pSB3K3 was also used to harbour the two-input AND gated biosensor constructs constructed following a similar three way ligation method (Fig. S1). The *hrpL* promoter from *Pseudomonas syringae* followed by a *gfp*, *rfp* or a *luxI* gene construct was carried on pSB4A3 (Shetty et al., 2008) (pSC101 ori, Amp^r^) using the same cloning strategy. The various RBS sequences (Table 1) for each gene construct were introduced with PCR primers if necessary (amplification utilized high-fidelity Phusion DNA polymerase from NEB and an Eppendorf Mastercycler gradient thermal cycler). All constructs were verified by DNA sequencing (Beckman Coulter Genomics at UK) prior to their use in the target cell strains. Primers were synthesised by Sigma Life Science. More information can be found in Fig. S1 Supporting methods, with plasmid maps describing some representative circuit constructs used.

### 2.3 Gene expression assay

Fluorescence outputs representing gene expression levels were measured by fluorometry at the whole cell population level. Cells grown in 96-well plates were monitored and assayed using a BMG FLUOstar fluorometer for repeated absorbance (OD_600_) and green fluorescence (485 nm for excitation, 520±10 nm for emission, Gain=1200, bottom reading) or red fluorescence (584 nm for excitation, 620±10 nm for emission, Gain=2000, bottom reading) readings (20 min/cycle). For fluorescent assay of the three-input biosensor comprising two cell consortia, the two cell populations were diluted from overnight culture to the same cell density at *OD*_600_=0.025 and mixed using the same volume of each cell culture prior to being loaded into microtitre plate wells. The mixed cell culture was then subjected to chemical exposure and incubation in the fluorometer. The normalised total fluorescence 10 h after induction was used for the three-input biosensor assay. This longer time growth allows for sufficient quorum sensing molecules synthesised by the first cell consortium to accumulate for activation of the second cell consortium under the specified conditions (see Fig. 4 legends).

### 2.4 Mathematical model and data analysis

**Fig. 2:**
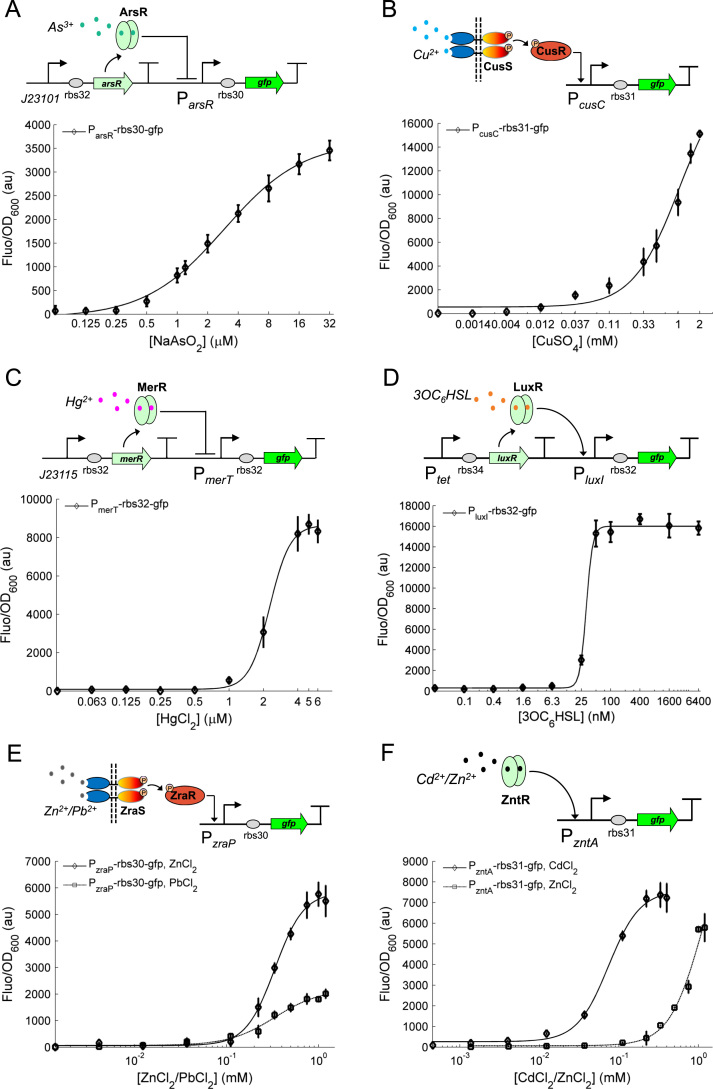
The design and characterization of a set of single-input cellular biosensors. (A) The arsenic sensor were characterised under various arsenite concentrations (0, 0.125, 0.25, 0.5, 1.0, 1.2, 2.0, 4.0, 8.0, 16.0, 32 μM NaAsO_2_). (B) The copper ion cellular sensor was characterised at various CuSO_4_ levels (0, 0.00137, 0.0041, 0.0123, 0.037, 0.11, 0.33, 0.5, 1.0, 1.5, 2.0 mM). (C) The mercury sensor was characterised under various HgCl_2_ levels (0, 0.0625, 0.125, 0.25, 0.5, 1.0, 2.0, 4.0, 5.0, 6.0 μM). (D) The quorum sensing signalling molecule sensor was characterised under various AHL levels (0, 0.098, 0.39, 1.56, 6.25, 25.0, 50, 100, 400, 1600, 6400 nM). (E) The zinc or lead sensor was characterised under various ZnCl_2_/PbCl_2_ levels (0, 0.0041, 0.0123, 0.037, 0.111, 0.222, 0.333, 0.5, 0.75, 1, 1.2 mM). (F) The cadmium or zinc sensor was characterised under various levels of CdCl_2_ (0, 0.00137, 0.0041, 0.0123, 0.037, 0.111, 0.222, 0.333, 0.4 mM) or ZnCl_2_ as the same as indicated in (E). Error bars, s.d. (*n*=4). a.u., arbitrary units.

**Fig. 3:**
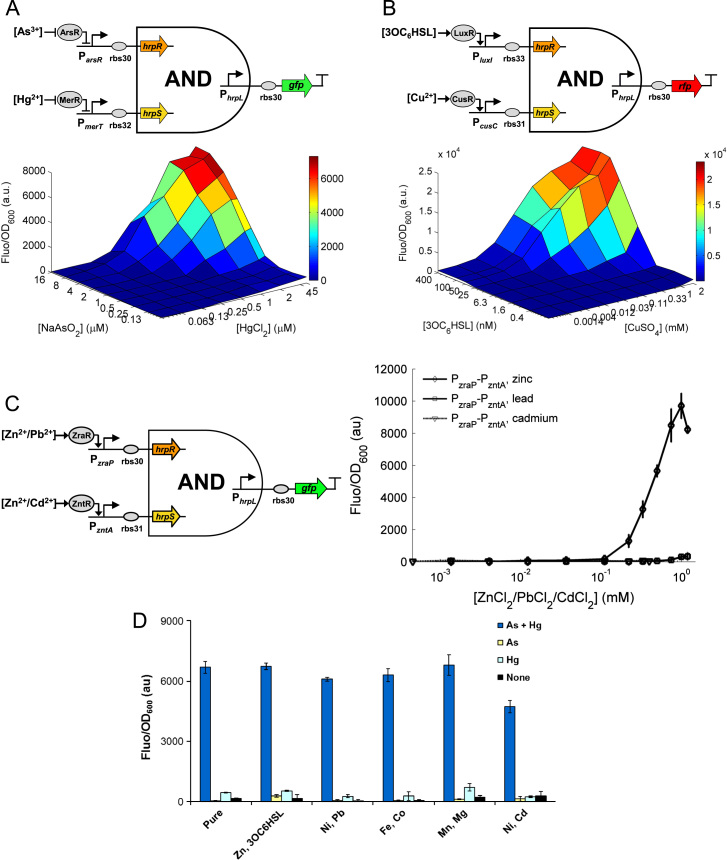
Two-input AND logic gated cellular biosensors. (A) The arsenite and mercury sensitive AND logic gated sensor was measured using 81 combinations of the two input metals (0, 0.125, 0.25, 0.5, 1.0, 2.0, 4.0, 8.0, 16.0 μM arsenite by 0, 0.0625, 0.125, 0.25, 0.5, 1.0, 2.0, 4.0, 5.0 μM mercury). (B) The 3OC_6_HSL and copper sensitive AND logic gated sensor was measured using 72 combinations of two input chemicals (0, 0.39, 1.56, 6.25, 25.0, 50, 100, 400 nM 3OC_6_HSL by 0, 0.00137, 0.0041, 0.0123, 0.037, 0.11, 0.33, 1.0, 2.0 mM CuSO_4_). (C) The AND logic gated zinc sensor was measured using various levels of ZnCl_2_ or PbCl_2_ (0, 0.0041, 0.0123, 0.037, 0.111, 0.222, 0.333, 0.5, 0.75, 1, 1.2 mM) or CdCl_2_ (0, 0.00137, 0.0041, 0.0123, 0.037, 0.111, 0.222, 0.333, 0.4 mM). (D) Behaviour of the AND gated sensor for arsenic and mercury (Fig. 3A) in the presence of other competing metals. The sensor responses are assayed under four logic combinations of the two target metal ions (16 μM NaAsO_2_ plus 2 μM HgCl_2_, 16 μM NaAsO_2_ only, 2 μM HgCl_2_ only, and no target metals) either in the absence of other metals (Pure) or in the presence of two other competing chemicals with each metal added at 0.1 mM and 3OC_6_HSL at 100 nM. Error bars, s.d. (*n*=4). In A and B, all data are the average of three repeats with variations less than 10% between biological replicates.

The transfer functions for the single-input and double-input AND gated sensors were derived from a steady state assumption (Wang et al., 2011). The resulting data (Fig. 2A–F) were fitted to a Hill function model for the environment responsive promoter steady state input-output response (transfer function) of the form (Alon, 2007; Wang et al., 2011; Zoltan et al., 2006):

(1) $$f([I])=k(\alpha +{\left[I\right]}^{n}/(K_{M}^{n}+{\left[I\right]}^{n}))$$

where [*I*] is the concentration of the inducer, *K*_*M*_ and *n* are the Hill constant and coefficient, respectively, relating to the promoter–regulator/inducer interaction, *k* is the maximum expression level due to induction, and *α* is a constant relating to the basal level of the promoter due to leaky expression (Table 2). A detailed derivation of the transfer functions is provided in Supplementary methods. The two dimensional data of the AND gated sensors (Fig. 3A and B) were used to parameterise the normalised transfer function model of the AND gate of the form:

(2) $$f([R],[S])=[G]/{\left[G\right]}_{\max}={\left([R]/K_{R}\right)}^{n_{R}}{\left([S]/K_{S}\right)}^{n_{S}}/((1+{\left([R]/K_{R}\right)}^{n_{R}})(1+{\left([S]/K_{S}\right)}^{n_{S}}))$$

The transfer function describes the normalised output of the AND gate as a function of the levels of the two activator proteins ([R] for HrpR, [S] for HrpS) at steady state. ${\left[G\right]}_{\max}$ is the maximum activity observed for the output. $K_{R}$, $K_{S}$ and $n_{R}$, $n_{S}$ are the Hill constants and coefficients for HrpR and HrpS, respectively (Supplementary methods, Fig. S3). The levels of the two activators were derived from the parameterised transfer functions of the two input promoters (Table 2) with the same RBSs as used in the AND gate construction. The nonlinear least square fitting function (cftool and sftool) in Matlab (MathWorks R2010a) was applied to fit the experimental data to parameterise the transfer function model. The fluorometry data of gene expression were first processed in BMG Omega Data Analysis Software (v2.10) and then exported into Microsoft Excel 2010 and Matlab for analysis. The medium backgrounds of absorbance and fluorescence were determined from blank wells loaded with LB media and were subtracted from the readings of sample wells. The sample fluorescence/OD_600_ ratio (Fluo/OD_600_) at a specific time was determined by subtracting the triplicate-averaged ratio corresponding to the negative control cultures (GFP-free) at the same time point. For the population-averaged assay by fluorometry, the fluorescence/OD_600_ 6 h growth post initial dilution and induction was treated as the output response of cells in the steady state during exponential growth.

## 3 Results and discussion

### 3.1 Design and construction of a set of single input cellular sensors

Through examination of the reported native environmental sensing abilities of different bacterial organisms, we first designed a set of whole cell based biosensors to detect contamination related molecules and heavy metal ions (arsenic, mercury, copper, zinc, cadmium and bacterial quorum sensing (QS) molecules) in the aqueous environment (Fig. 2). These biosensors were designed either by harnessing the natural signalling mechanisms of the host cells, such as the *E. coli* genetic resistant modules against high concentrations of arsenite (Diorio et al., 1995), copper (Munson et al., 2000), zinc (Leonhartsberger et al., 2001) and cadmium ions (Binet and Poole, 2000), or by utilising synthetic sensory pathways from other bacteria such as the mercury sensitive MerR protein and its cognate regulated promoter *P*_merT_ from *Shigella flexneri* R100 plasmid (Misra et al., 1985), in addition to the QS signalling molecule 3OC_6_HSL sensitive LuxR and its target *P*_luxI_ promoter from *Vibrio fischeri* (Stevens and Greenberg, 1997). A GFP (green fluorescent protein) mutant with fast maturation time was used as the output reporter to indicate the levels of these chemicals.

Fig. 2A shows the architecture of the genetically encoded arsenic sensor. The synthetic sensor circuit comprises the arsenite/arsenate sensitive ArsR repressor continuously expressed from a constitutive promoter J23101 and its cognate *P*_arsR_ promoter fused to a *gfp* reporter. When arsenic is absent, the *P*_arsR_ promoter is bound and repressed by ArsR resulting in low fluorescent output. The circuit produces high fluorescence when arsenic exists and binds with ArsR to relieve its repression on the target promoter. The spatial separation of the repressor and its cognate promoter in this design is different from that in their native context in order to reduce the sensor background basal expression level. The dose response of this arsenic sensors (Fig. 2A) shows that the sensor output is proportional to the arsenic levels in the media with detection limit as low as 0.1 μM. The characterization data were fitted to the derived transfer function model of the sensor (see Section 2) and the fitted model parameters are shown in Table 2.

Fig. 2B shows the design and characterisation of the copper responsive sensor. The sensor circuit comprises simply the *P*_cusC_ promoter fused to the *gfp* reporter by utilizing the *E. coli* native CusSR two component signal transduction pathway (Munson et al., 2000). The histidine sensor kinase CusS is embedded in the cell membrane to sense extracellular copper ions and can phosphorylate its response regulator CusR under high Cu^2+^ conditions. The phosphorylated CusR can then activate the target *P*_cusC_ promoter to turn on the GFP production. Therefore the output fluorescence level is in proportion to the copper ion level in the environment. The dose response curve of the sensor (Fig. 2B) shows that the sensor can detect Cu^2+^ between micro- and milli-molar range with a detection limit as low as 12 μM. The characterised dose responses were next fitted to the transfer function model (Eq. (1), parameters shown in Table 2).

Fig. 2C and D shows the design and characterisation of the mercury and QS molecule 3OC_6_HSL sensors respectively. These two sensors were designed using the orthologous sensory proteins MerR and LuxR and their cognate target promoters *P*_merT_ and *P*_luxI_ from other bacteria. The two sensor circuits have similar genetic architectures: the receptor proteins are continuously expressed from a constitutive promoter (J23115 or *P*_tet_) with their cognate regulated promoters fused to the *gfp* reporter. The two sensory proteins can bind specifically to their cognate mercury and 3OC_6_HSL molecules and the bound complexes then activate their target promoters to turn on the GFP reporter production. The characterised dose response curves of the two sensors (Fig. 2C and D) show that they have similar digital-like responses with a steep transition from the OFF state to the ON state. The fitted Hill function models (Table 2) also indicate that the two sensors have high cooperative Hill coefficients (4.84 for the mercury sensor and 6.64 for the 3OC_6_HSL sensor) but with distinctly different Hill constants (2.3 μM for mercury and 31.6 nM for 3OC_6_HSL), which might be due to the different binding affinities between the two receptor proteins and their cognate signalling molecules.

Fig. 2E and F shows the design and characterisation of the zinc and cadmium responsive sensors. The zinc sensor comprises of the *P*_zraP_ promoter fused to the *gfp* reporter by utilizing the *E. coli* native ZraSR two component signal transduction pathway (Leonhartsberger et al., 2001). The zinc responsive sensor kinase ZraS senses extracellular metal ions and can phosphorylate its response regulator ZraR under high Zn^2+^ or Pb^2+^ conditions. The phosphorylated ZraR can then activate the target *P*_zraP_ promoter leading to GFP production. The cadmium sensor comprises the *P*_zntA_ promoter fused to the *gfp* reporter by utilizing the *E. coli* native metal exporting ATPase pathway for Cd^2+^ or Zn^2+^ (Binet and Poole, 2000). The dose response curves show that the zinc sensor can sense both Zn^2+^ and Pb^2+^ though with a decreased dynamic range for Pb^2+^ (Fig. 2E) while the cadmium sensor can sense both Cd^2+^ and Zn^2+^ though at a less sensitive level for Zn^2+^ in the micro- and milli-molar range (Fig. 2F). The characterised dose responses were fitted to the transfer function model (Eq. (1), Table 2).

The input-output dose responses show that the output strengths of these sensors are different, i.e., some regulated promoters like *P*_merT_ and *P*_luxI_ are strong while others like *P*_arsR_ and *P*_cusC_ are relatively weak. Thus we used different RBS (ribosome binding site) to tune the maximum fluorescence output of these sensors by regulating the translational initiate rate of the *gfp* reporter (Table 1). Normally, using strong RBS can increase the sensitivities of the sensor but will slightly increase the background basal level. Because these single input sensors will also be connected to the genetic logic gates in the next step, strong RBSs were used for characterizing arsenic and copper sensors and weak RBSs were used for characterizing mercury and 3OC_6_HSL sensors (Fig. 2) in order to balance their maximum output and default leaky levels. The detection thresholds of these sensors can also be tuned by varying the receptor protein levels in the cytoplasm. Typically, high level of the repressor receptor protein (e.g., ArsR) in the cell will decrease the sensitivity of the sensor while high level of the activator receptor protein (e.g., LuxR) will increase the sensitivity of the sensor. The constructed synthetic sensors have been developed to exhibit very high selectivity only for their target signalling molecules but with almost no or little recognition specificity for a broad range of non-target molecules, as indicated in Fig. S2, with the exception of moderate multiple metal responses in the *P*_zraP_ (responsive to Zn^2+^ and Pb^2+^) and *P*_zntA_ (responsive to Cd^2+^, Zn^2+^ and Hg^2+^) sensors.

### 3.2 Design and construction of modular double-input AND logic gated sensors

Based on the single input cell-based biosensors constructed, we next sought to construct an advanced biosensor that can detect two input signals at the same time and integrate them following a designed logic before producing the output response.

To design a logic AND-gated cellular biosensor, we connected two transcriptional inputs of the single input sensors to a modular and orthogonal genetic AND gate we have engineered in *E. coli* recently (Wang et al., 2011) and used *gfp* or *rfp* as the output readout. The modular two-input AND gate comprises two heterologous genes, *hrpR* and *hrpS*, and one σ^54^-dependent output promoter, *hrpL*, from the *hrp* (hypersensitive response and pathogenecity) regulatory system of the plant pathogen *P. syringae* (Hutcheson et al., 2001; Wang et al., 2011). The *hrpR* and *hrpS* encode two regulatory enhancer binding proteins that act synergistically by forming a heteromeric protein complex to co-activate the tightly regulated *hrpL* promoter (Jovanovic et al., 2011). Both the inputs and output of the AND gate were designed to be promoters to facilitate their connection to different upstream and downstream transcriptional modules. Due to this modularity, the inputs can be rewired to different input sensors and the output can be used to drive various cellular responses.

Fig. 3A shows the schematic design of a two-input AND gated biosensor that can detect both arsenic and mercury ions with *gfp* as the output indicator. The synthetic sensor circuit comprises the arsenic and mercury sensors connected to the inputs of the modular AND gate to drive *hrpR* and *hrpS* respectively and the *gfp* fused to the *hrpL* output promoter of the AND gate. The constructed sensor behaviour was then systematically characterized in a 96 well microtitre plate by measuring the output responses under 81 combinations of the two input chemical inducers of varying concentrations. Fig. 3A shows that the device behaves as expected, with clear digital-like logic AND characteristics: the sensor output is high only when both arsenic and mercury are above a certain threshold, similar to that of the single input sensors, sufficient to activate the two inputs of the genetic AND gate.

Fig. 3B shows the schematic design of a second double-input AND gated biosensor that can detect and integrate Cu^2+^ and the quorum sensing molecule 3OC_6_HSL. Using a similar architecture, the sensor circuit comprises the copper and 3OC_6_HSL sensors connected to the AND gate inputs and the *rfp* connected to the AND gate output promoter. The device was systematically characterized using 72 combinations of the two dimensional chemical inductions. The responses (Fig. 3B) show that the sensor behaves much like a digital logic AND gate device. The sensor produced a very high fluorescent output when both Cu^2+^ and 3OC_6_HSL reach a certain threshold in the media with nearly zero activities at default off state.

Fig. 3C shows the design and characterization of a further double-input AND gated biosensor that can distinguish between Zn^2+^ and Pb^2+^ or Cd^2+^. The sensor circuit employs *P*_zraP_ (responsive to Zn^2+^ and Pb^2+^) and *P*_zntA_ (responsive to Cd^2+^ and Zn^2+^) as the sensory inputs to the AND gate and the *gfp* as the output reporter. Because both of the two sensory inputs have to be activated in order to generate the fluorescent output, this AND logic gated sensor is only responsive to Zn^2+^ but not Pb^2+^ or Cd^2+^ in conditions containing only a single contaminant of these metals. The dose response curves of the sensor to Zn^2+^, Pb^2+^ or Cd^2+^ confirm that the sensor is not only highly selective to zinc but also has an increased absolute fluorescent output, i.e. signal-to-noise ratio.

The successful engineering of the double-input AND gated biosensors demonstrates the possibility of using synthetic sensory and gene regulatory circuits to rewire host cell native signalling and so to generate novel customized behaviour. The integrated circuit behaviour is also predictable by following a forward engineering approach with well characterised component parts and modules. Due to the modular architecture of the system, different versions of the double input biosensors can be readily constructed either by rewiring the inputs to new sensors or using different modular logic gates such as NAND and NOR gates to have desired logic gated responses. One possible feature and a potential drawback of using genetic cascades to process the multiple input signals in these sensors is that it might lead to slightly longer response time (as compared to the case without a second layer of genetic circuit regulation) due to the extra time needed for the downstream gene expression and regulation. Nevertheless, utilizing genetic logic gates for biosensing also bring clear advantages. For example, as exemplified by the zinc responsive AND gated sensor (Fig. 3C), the genetic AND gate can function as a genetic filter (it filters out non specific input signals enhancing the sensing selectivity of the sensor) as well as functioning as a genetic amplifier (it increases the sensor output readout level and thus the sensing sensitivity, i.e., the signal-to-noise ratio). To evaluate the robustness of these synthetic cell-based sensors, as well as to simulate the real hazard environment, the behaviour of the AND gated sensor in Fig. 3A was measured under a range of media conditions containing various other competitive metals at sub millimolar concentrations. The results (Fig. 3D) show that the arsenic and mercury responsive sensor behaves stably across the five simulated environmental conditions in which two other chemicals or metals were added to the cell medium.

### 3.3 Design and construction of a three-input cell consortium based biosensor

Despite the advanced capabilities of the cellular sensors comprising only single cell type populations, systems containing multiple cell type populations might lead to more complex and high level functions through intercellular communication and cooperation (Brenner et al., 2007; Prindle et al., 2012; Weber et al., 2007). Particularly, single sensor systems that can detect and integrate more than two signals in parallel are necessary when high specificity and accuracy of response is required in certain situations such as in disease diagnosis.

**Fig. 4:**
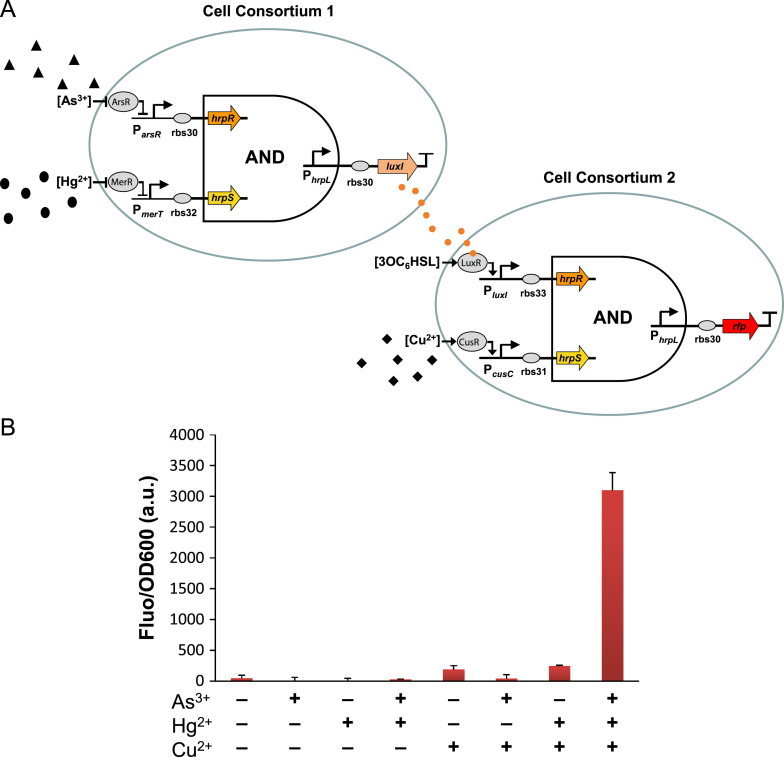
A three-input cellular biosensor coupling two cell consortia via intercellular communication. (A) Each cell consortium was programmed with an AND logic circuit that can detect and integrate two specific input signals. The output of the first consortium – 3OC_6_HSL, synthesized by the LuxI enzyme, functions as one of the signalling inputs to the second consortium. (B) The device was tested under 8 logic combinatorial conditions of 8 μM NaAsO_2_, 2 μM HgCl_2_, 0.33 mM CuSO_4_. The data were acquired 10 h post chemical exposure. Error bars, s.d. (*n*=3).

Built on the two double-input sensors above, a triple-input AND gated biosensor comprising two cell consortia was constructed by coupling two cascaded cell populations, each itself functioning as a double-input sensor (Fig. 4A). The first cell consortium in the system is a double-input AND gated sensor that can sense arsenic and mercury as described in Section 3.2 but using the *luxI* gene as the output instead of *gfp* now. The LuxI enzyme is the synthase for the QS molecule 3OC_6_HSL which can freely diffuse through the cell membrane into the surrounding media. The second cell consortium is the double-input AND gated sensor as shown in Fig. 3B which can sense 3OC_6_HSL and Cu^2+^ with *rfp* as the output readout. Thus the output signal of the first consortium functions as one of the inputs of the second consortium. The integrated system comprising the two consortia results in an AND gated three input biosensor that can detect and integrate arsenic, mercury and copper ion levels. Fig. 4B shows the sensor responses under eight logic combinations of the three input signals (8 μM NaAsO_2_, 2 μM HgCl_2_ and 0.33 mM CuSO_4_) by inoculating the same number of cells of each strain in one pool. The sensor behaves indeed as a triple input logic AND gate device in that a high red fluorescent response was seen only when all the three metal ions exist at high levels in the culture, illustrating the combinatorial logic character of the system.

This synthetic system is the first modular cellular sensor constructed so far that can detect and integrate three customizable environmental signals in parallel. The system can be readily adapted to sense other signals by exchanging the sensory inputs of the component circuit modules and strains. Due to its modular architecture, the system also has the potential to be scaled up to detect more than three environmental signals, for example by utilizing more orthogonal cell communication modules such as the LasI/LasR and RhlI/RhlR quorum sensing systems in *P. aeruginosa* (Brenner et al., 2007). One potential future research focus is to vary the inoculation ratio and cell densities of the two cell populations to investigate the impact of these parameters on the system behaviour. Owing to the multiple layers of genetic cascades within these intercellular systems, much longer response time might be needed for synthesizing the signalling molecules to achieve efficient cell communication and activation of the downstream genetic modules.

## 4 Conclusions

A set of scalable microbe-based biosensors were constructed to detect and integrate both single and multiple environmental contamination associated signals. The synthetic cellular sensors are designed to follow a modular architecture comprising exchangeable genetic sensory, signal processing and integration, and actuation modules. These *E. coli*-based biosensors were programmed using signalling sensory modules either from native two-component signal transduction pathways or synthetic signalling sensors from other bacteria. The sensory modules were then wired to a genetic logic AND gate to distinguish As^3+^, Hg^2+^, Cu^2+^, Zn^2+^ and the 3OC_6_HSL quorum sensing molecule and combinations thereof with quantitative fluorescent output. Importantly, such genetic logic gates can function as a biological filter and an amplifier to enhance the sensing selectivity and sensitivity of cell-based biosensors. Additionally, a two cell population, *E. coli* consortium-based biosensor was engineered to detect and integrate three environmental signals via intercellular communication. Customization to sense other environmental signals, by exchanging the genetic sensory modules, is readily possible. Equally, customization to obtain other novel biosensor outputs is also possible. For example, by connecting the sensor output promoter to direct the synthesis of an electron mediator, a direct digital electrical current could be generated in a bio-electrochemical system (Li et al., 2011). Here we have demonstrated how a modular cell-based biosensor can be engineered predictably using exchangeable synthetic gene circuit modules to sense and integrate multiple-input signals. Our studies illustrate some of the key practical design principles required for their future application in biosensing for a broad array of environmental and healthcare areas.

## Figures and Tables

**Table 1:** Ribosome binding sites used for characterising sensor dose responses.

**Identifier**^a^	**Sequence of RBS**^b^	**Observed strength**
rbs30	TCTAGAGATTAAAGAGGAGAAATACTAG**ATG**	very strong
rbs31	TCTAGAGTCACACAGGAAACCTACTAG**ATG**	strong
rbs32	TCTAGAGTCACACAGGAAAGTACTAG**ATG**	weak
rbs33	TCTAGAGTCACACAGGACTACTAG**ATG**	very weak

a The RBS sequences are from the Registry of Standard Biological Parts (http://partsregistry.org) and are reported to have distinct strengths (Wang et al., 2011).

b Sequences of RBS are underlined and start codons are in bold.

**Table 2:** The best fits for characterised dose responses of the single-input biosensors with 95% confidence.

**Sensor reporter**	**Target**	***k*** (a.u.)	***n***	***K***_***M***_ (μM)	***α***	***R***^**2**^
*P*_arsR_-rbs30-gfp	Arsenic	3719±431	1.115±0.251	2.899±0.632	−0.0185±0.0438	0.9971
*P*_cusC_-rbs31-gfp	Copper	2.0e4±1.383e4	1.343±0.868	1.04e3± 1.18e3	0.0277±0.0503	0.9883
*P*_merT_-rbs32-gfp	Mercury	8563±594	4.84±2.166	2.267±0.191	0.0106±0.0241	0.9983
*P*_luxI_-rbs32-gfp	3OC_6_HSL	1.571e4±0.056e4	6.635±2.011	31.64e−3±2.57e−3	0.0188±0.0242	0.9986
P_zraP_-rbs30-gfp	Zinc	5780±392	2.762±0.585	0.335e3±0.027e3	0.0107±0.0270	0.9979
P_zraP_-rbs30-gfp	Lead	2114±548	1.749±0.933	0.334e3± 0.123e3	0.0306±0.0668	0.9883
P_zntA_-rbs31-gfp	Cadmium	7326±579	2.192±0.581	0.074e3±0.012e3	0.0359±0.0375	0.9980
P_zntA_-rbs31-gfp	Zinc	1.12e4±1.62e4	2.085±1.641	1.117e3±1.513e3	0.0059±0.0391	0.9821

## Footnotes

Appendix A Supplementary data associated with this article can be found in the online version at doi:10.1016/j.bios.2012.08.011.
