# Supplementary material for: A modular cell-based biosensor using engineered genetic logic circuits to detect and integrate multiple environmental signals
Source: Biosens Bioelectron. 2013 Feb 15;40(1):368–76. doi: 10.1016/j.bios.2012.08.011 (PMC3507625; doi:10.1016/j.bios.2012.08.011)
Supplement: Supplementary file 1 — Supplementary Material [file mmc1.doc]

**A B**

**C**

**Fig. S1**. **Plasmid maps showing some representative circuit constructs used in this study.** (**A**) The plasmid for encoding the single input arsenic sensor with *gfp* as the output (Fig. 2A). (**B**) The plasmid for encoding the single input mercury sensor with *gfp* as the output (Fig. 2C). (**C**) The two plasmids for encoding the AND gated biosensor that detects and integrates arsenic and mercury (Fig. 3A). The left is the input plasmid for signal sensing and the right is the output plasmid for signal integration and GFP production.

**A**


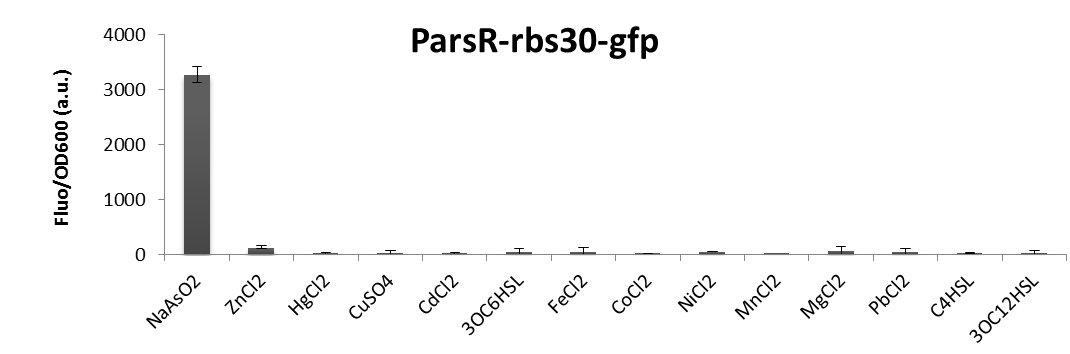


**B**


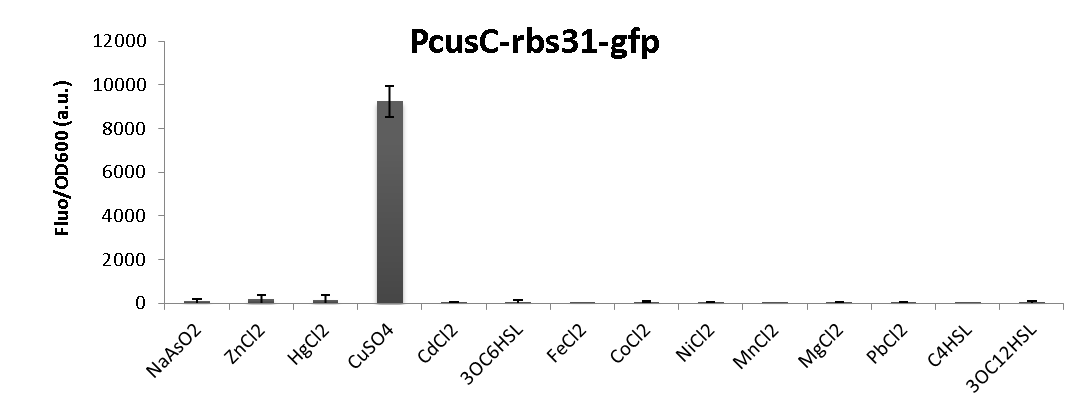


**C**


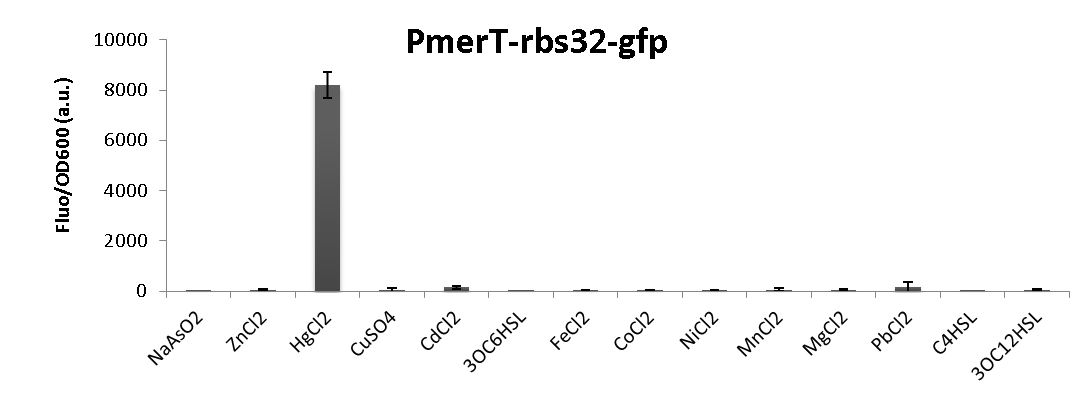


**D**


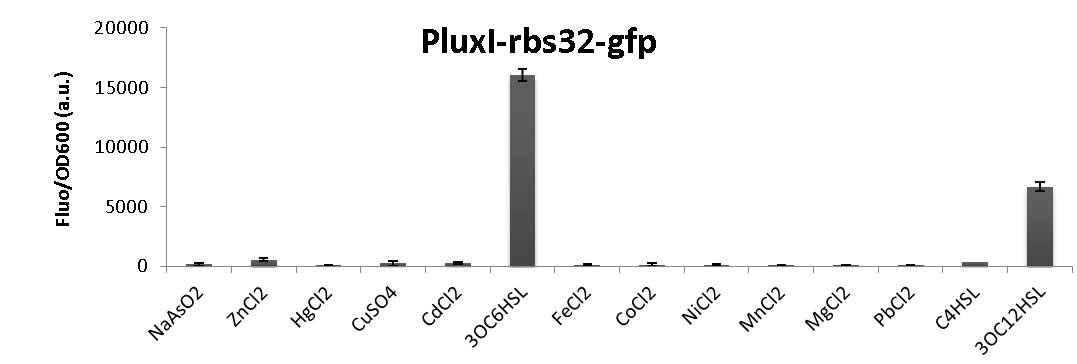


**E**


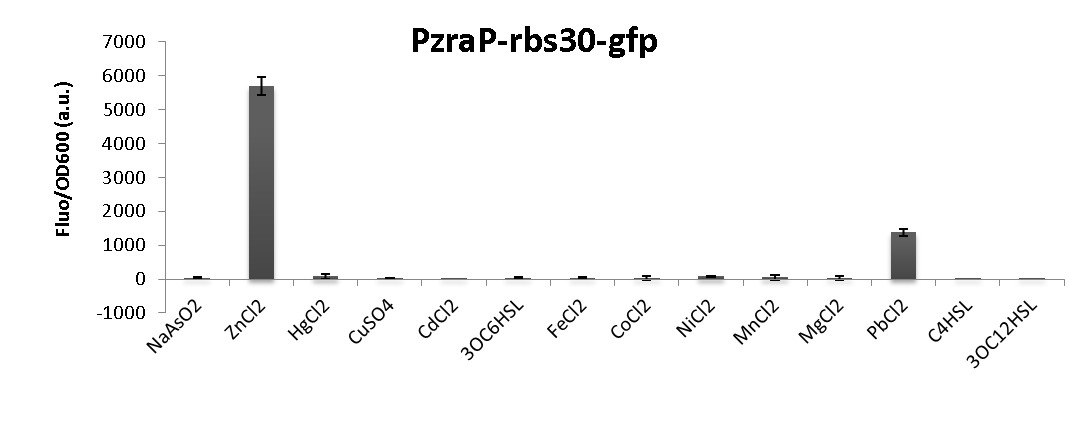


**F**


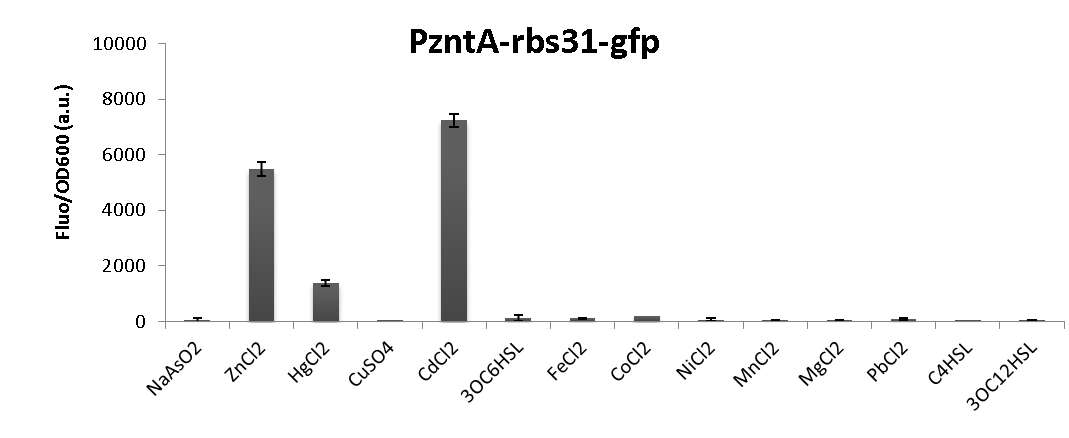


**Fig. S2**. The specificity of the single-input cellular biosensors. *E. coli* TOP10 strain harbouring different sensors were cultured separately in LB containing various chemicals at 37 °C under oxic conditions. NaAsO_2_ was added at 16 μM, HgCl_2_ at 4 μM, ZnCl_2_ and CuSO_4_ at 1 mM, CdCl_2_, FeCl_2_, CoCl_2_, NiCl_2_, MnCl_2_, MgCl_2_ and PbCl_2_ at 0.4 mM, 3OC_6_HSL and 3OC_12_HSL at 400 nM and C_4_HSL at 100 μM. The fluorescent data were acquired 6 hrs post induction. The activities shown are for the arsenic (**A**), copper (**B**), mercury (**C**), quorum sensing molecule (**D**), zinc (**E**) and cadmium (**F**) responsive sensors respectively. Error bars, s.d. (n = 3).

**Supplementary methods**

**Mathematical modelling and data fitting**

Computational models were developed for the single-input bacterial promoter reporters and the genetic AND logic gated sensors. We focus on the average behaviour of the *E. coli* population to demonstrate the performance of the engineered sensors at steady state. The ODEs-based deterministic model was used for modelling gene regulation and expression. The following describes the derivation of the transfer function (TF) for each genetic module and the experimental data fitting to these models.

**1. Deriving transfer function of the environment responsive promoter-based sensor**

The diagram above shows the exemplar architecture of the environment-responsive promoter-based sensors used in this study. The promoter P_1_ is negatively regulated by its constitutively expressed repressor R_1_ and is responsive to exogenous inducer I_1_ to activate transcription of downstream reporter gene *G*. The reporter gene expression can be modelled by (Alon, 2007; Zoltan et al., 2006):

$\frac{d[G]}{dt}\text{ = }\alpha\cdot k_{1}+\frac{k_{1}\cdot[I_{1}]^{n}}{[I_{1}]^{n}+K_{1}^{n}}-d\cdot[G]$ (S1)

where $\alpha\cdot k_{1}$ is the basal constitutive activity of the promoter, $k_{1}\cdot[I_{1}]^{n}/([I_{1}]^{n}+K_{1}^{n})$ is the activity due to cooperative transcription activation by assuming the concentration of the repressor is constant to model the effect of varying the concentration of the inducer $I_{1}$, and $d\cdot[G]$is the constitutive degradation activity of protein G. $K_{1}$ and $n$ are the Hill constant and coefficient relating to the promoter-regulator/inducer interaction, $k_{1}$ is the maximum expression rate due to induction and $\alpha$ is a constant relating to the promoter basal level due to leakage (0 ≤ $\alpha$ < 1), and $d$ is the degradation rate of G.

The steady state solution of equation S1 is given by

$f([I_{1}])\text{ = }[G]_{ss}\text{ = }k(\alpha+[I_{1}]^{n}/(K_{1}^{n}+[I_{1}]^{n}))$ (S2)

in which $k=k_{1}/d$ represents the maximum expression level due to induction. Equation S2 gives the reporter protein level at steady state for the inducible promoter P_1_ and is also the TF of P_1_. We used this TF to fit the characterisation data of the single-input sensors using the nonlinear least square fitting function (cftool) in Matlab. The best fit coefficients (with 95% confidence bounds otherwise fixed at bound) are listed in Table 2.

**2. Deriving transfer function of the AND gated sensor**

The diagram above shows the architecture of the AND gated sensor in this study. *hrpL* promoter is synergistically co-activated by the hetero proteins HrpR and HrpS, which mimics the logic AND function Based on the known mechanism underlying this hetero-regulated module, both the bacterial enhancer-binding proteins are required to bind the UAS (upstream activation sequence) of *hrpL* to remodel the conformation of σ^54^-RNAP-*hrpL* close complex to an open one for the transcriptional activation. The normalised AND gate TF is described by the product of two Hill function curves (Alon, 2007):

(S3)

in which $K_{R}$, $K_{S}$ and $n_{R}$, $n_{S}$ are the Hill constants and coefficients for HrpR and HrpS. $[R]_{ss}$ and $[S]_{ss}$ are the steady levels of HrpR and HrpS, whose levels are under the control of two separate environment-responsive promoters P_1_ and P_2_ as indicated by equation S2. is the maximum output level of the AND gate at steady state.

The TF was parameterised by fitting to the experimental data of the AND gate sensors (Fig. 3A-B) by nonlinear least square optimisation (sftool) in Matlab. Fig. S3A-B show the linear correlation between the fitted and experimentally characterised responses of the AND gate sensors.

**A B**

**
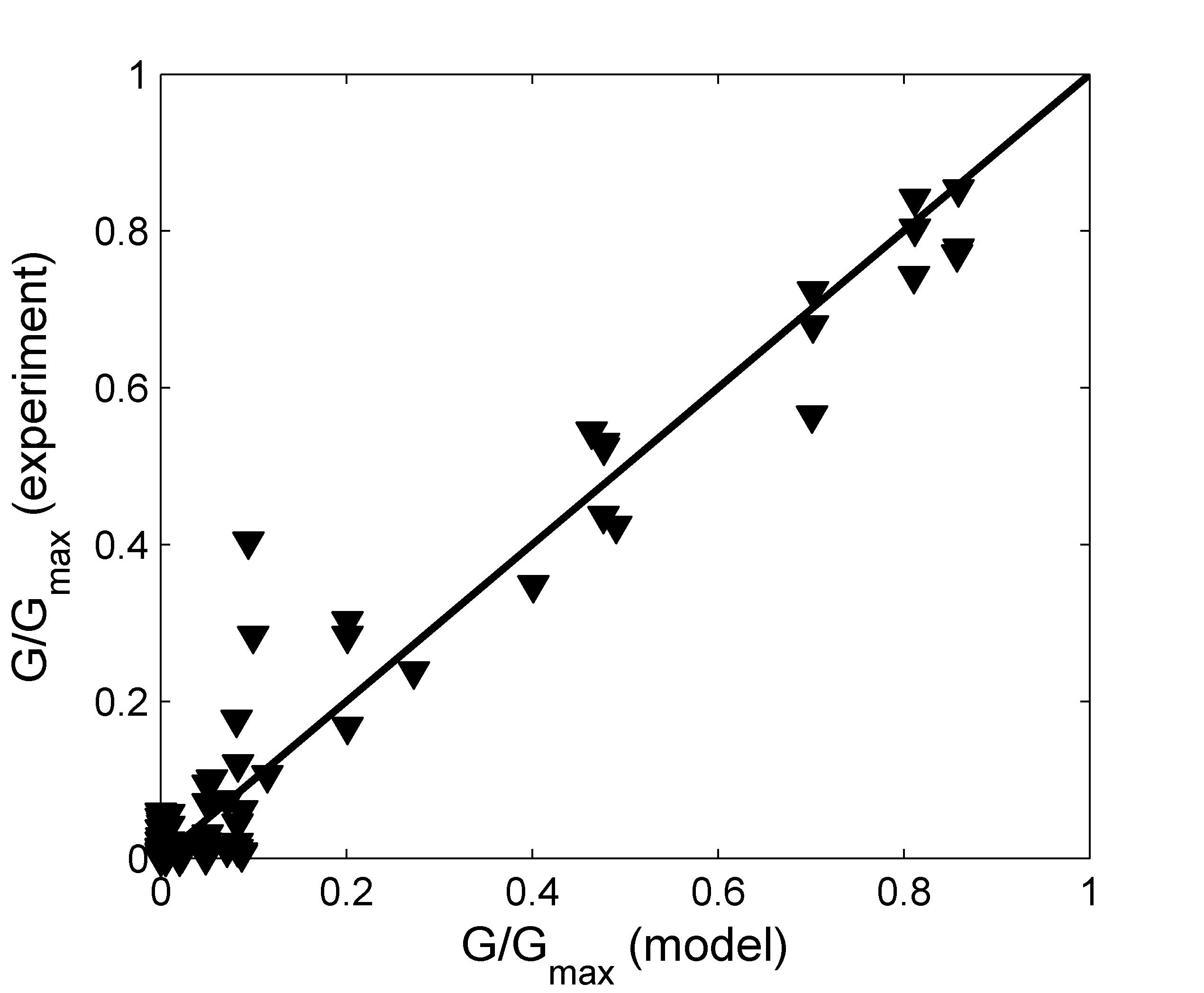

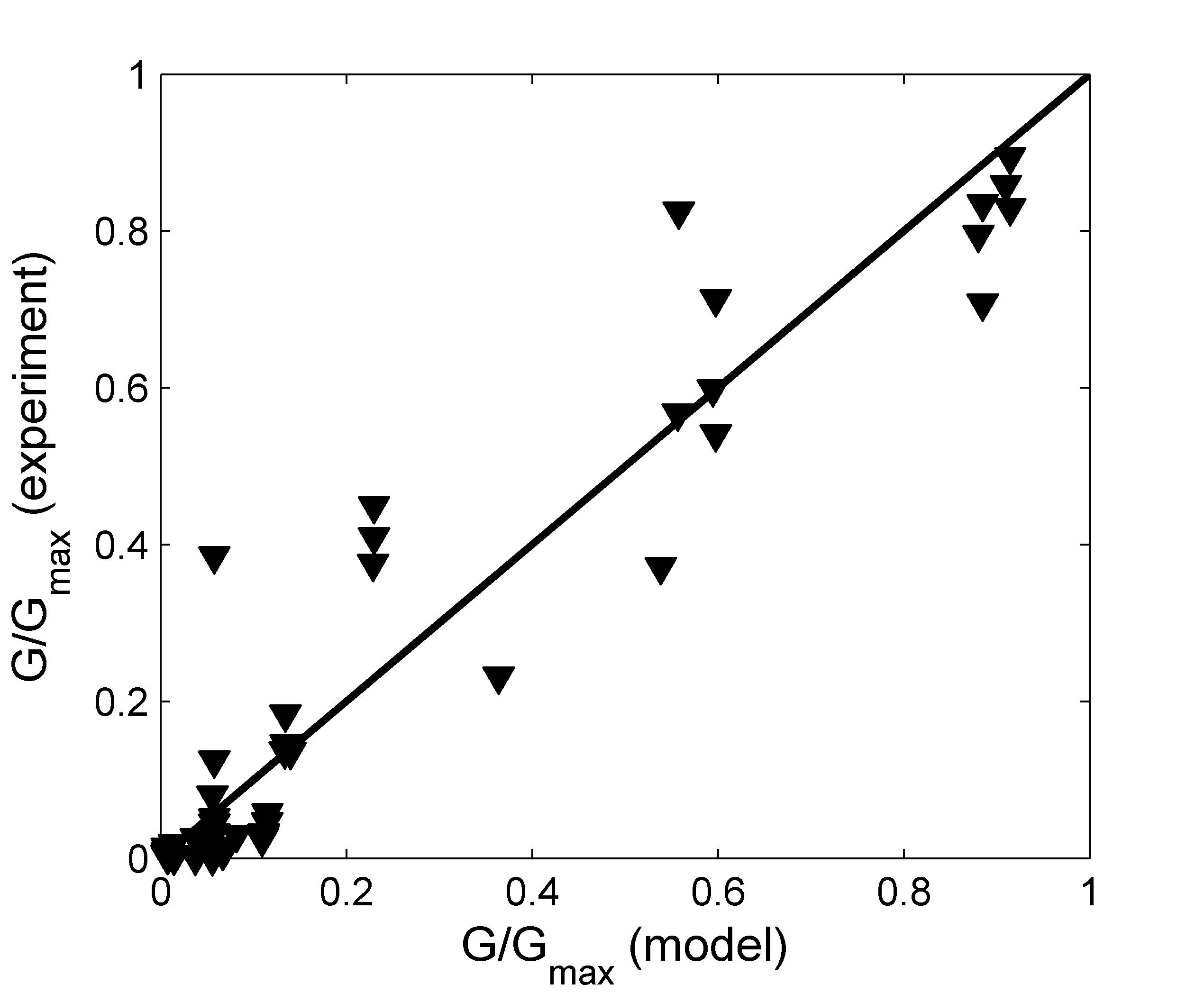
**

**Fig. S3.** The correlation between the fitted and experimentally characterised responses of the AND gate sensors. (**A**) The data fitting of the AND gated sensor shown in Fig. 3A to equation (S3) yield: $K_{R}$ = 1536 ± 464, $K_{S}$ = 222.8 ± 29.8, $n_{R}$ = 2.039 ± 0.679 and $n_{S}$ = 2.422 ± 0.527 with $[G]_{\max}$ = 8561 au with the Pearson correlation coefficient of 0.9722. (**B**) The data fitting of the AND gated sensor shown in Fig. 3B to equation (S3) yield: $K_{R}$ = 251.9 ± 107.7, $K_{S}$ = 1175 ± 173, $n_{R}$ = 1.301 ± 0.462 and $n_{S}$ = 2.657 ± 0.693 with $[G]_{\max}$ = 2.24e4 au with the Pearson correlation coefficient of 0.9632.

**Supplementary references**

Alon, U., 2007. An Introduction To Systems Biology: Design Principles Of Biological Circuits. Chapman & Hall/CRC, London.

Zoltan, S., Jörg, S., Vipul, P., 2006. System Modeling In Cell Biology: From Concepts To Nuts And Bolts. The MIT Press, Boston.
